# Cas9/AAV9-Mediated Somatic Mutagenesis Uncovered the Cell-Autonomous Role of Sarcoplasmic/Endoplasmic Reticulum Calcium ATPase 2 in Murine Cardiomyocyte Maturation

**DOI:** 10.3389/fcell.2022.864516

**Published:** 2022-04-01

**Authors:** Junsen Lin, Zhan Chen, Luzi Yang, Lei Liu, Peng Yue, Yueshen Sun, Mingming Zhao, Xiaoling Guo, Xiaomin Hu, Yan Zhang, Hong Zhang, Yifei Li, Yuxuan Guo, Erdan Dong

**Affiliations:** ^1^ Peking University Health Science Center, School of Basic Medical Sciences, The Institute of Cardiovascular Sciences, Key Laboratory of Molecular Cardiovascular Science of Ministry of Education, Beijing Key Laboratory of Cardiovascular Receptors Research, Beijing, China; ^2^ Key Laboratory of Birth Defects and Related Diseases of Women and Children of Ministry of Education (MOE), Department of Pediatrics, West China Second University Hospital, Sichuan University, Chengdu, China; ^3^ State Key Laboratory of Complex Severe and Rare Diseases, Department of Cardiology, Peking Union Medical College Hospital, Chinese Academy of Medical Science and Peking Union Medical College, Beijing, China; ^4^ Department of Medical Research Center, Peking Union Medical College Hospital, Chinese Academy of Medical Science and Peking Union Medical College, Beijing, China; ^5^ Department of Cardiology and Institute of Vascular Medicine, Peking University Third Hospital, National Health Commission of China (NHC) Key Laboratory of Cardiovascular Molecular Biology and Regulatory Peptides, Beijing Key Laboratory of Cardiovascular Receptors Research. Beijing, China; ^6^ Basic Medical Research Center, The Second Affiliated Hospital and Yuying Children’s Hospital of Wenzhou Medical University, Wenzhou, China

**Keywords:** cardiomyocyte maturation, Cas9/AAV9-mediated somatic mutagenesis, sarcoplasmic/endoplasmic reticulum calcium ATPase 2, heart failure, genetic mosaic analysis

## Abstract

Sarcoplasmic/endoplasmic reticulum calcium ATPase 2 (SERCA2) is a key player in cardiomyocyte calcium handling and also a classic target in the gene therapy for heart failure. SERCA2 expression dramatically increases during cardiomyocyte maturation in the postnatal phase of heart development, which is essential for the heart to acquire its full function in adults. However, whether and how SERCA2 regulates cardiomyocyte maturation remains unclear. Here, we performed Cas9/AAV9-mediated somatic mutagenesis (CASAAV) in mice and achieved cardiomyocyte-specific knockout of *Atp2a2*, the gene coding SERCA2. Through a cardiac genetic mosaic analysis, we demonstrated the cell-autonomous role of SERCA2 in building key ultrastructures of mature ventricular cardiomyocytes, including transverse-tubules and sarcomeres. SERCA2 also exerts a profound impact on oxidative respiration gene expression and sarcomere isoform switching from *Myh7/Tnni1* to *Myh6/Tnni3*, which are transcriptional hallmarks of cardiomyocyte maturation. Together, this study uncovered a pivotal role of SERCA2 in heart development and provided new insights about SERCA2-based cardiac gene therapy.

## 1 Introduction

Animal development is associated with the proportional increase of heart functions. In embryos, heart functions are improved through the establishment of normal anatomical structures as well as the differentiation and proliferation of the working cardiomyocytes. At the perinatal stage, cardiac morphogenesis nearly finishes and the cardiomyocyte number stops increasing. To further enhance the cardiac output, cardiomyocytes undergo maturation (Guo and Pu, 2020), a process characterized by fundamental gene expression and ultrastructural changes that greatly enhance cardiomyocyte robustness.

Calcium handling is central to cardiomyocyte functions, and therefore a major gauge of cardiomyocyte maturity (Guo and Pu, 2020). At the start of cardiomyocyte contraction, electrical signals trigger plasma membrane depolarization and activate L-type calcium channels (LTCCs). LTCC activation leads to ryanodine receptor 2 (RYR2) opening and the rapid calcium release from the sarcoplasmic/endoplasmic reticulum (SR/ER) lumen to the cytoplasm. The rise of cytoplasmic calcium concentration activates sarcomere shortening and cell contraction. At the end of cardiomyocyte contraction, cytoplasmic calcium is actively depleted for sarcomere relaxation. This process is mainly driven by the SR/ER calcium ATPase 2 (SERCA2, coded by *Atp2a2* gene), an ATP-consuming pump spanning the SR/ER membrane and transporting calcium back to the SR/ER lumen.

Two major cellular changes enhance the calcium handling capacity in cardiomyocyte maturation. The first change is characterized by the gradual increase of major calcium handling molecules, such as RYR2 and SERCA2 (Liu et al., 2002). In addition, cardiomyocytes develop specialized subcellular ultrastructures such as the transverse tubules (T-tubule). T-tubules are invaginations of plasma membrane that facilitate action potential propagation from cell surface to cell interior and boost rapid and synchronous calcium release throughout the intracellular space (Brette and Orchard, 2003). In mice, T-tubules form at the second and third weeks after birth (Chen et al., 2013).

The above developmental changes about calcium handling is of particular interest because of their tight association with cardiac pathogenesis. SERCA2 reduction is well studied to contribute to heart failure. Enhancing SERCA2 expression or activity is a promising strategy in the gene therapy for heart failure (Hajjar et al., 1998; Del Monte et al., 2001; Sakata et al., 2007; Kawase et al., 2008). T-tubule disruption is also reported as a hallmark of heart failure. Stabilizing or rebuilding T-tubules is speculated as a promising strategy for cardiac therapy as well (Guo et al., 2013; Ibrahim and Terracciano, 2013). Collectively, these studies indicated that rebuilding the mature calcium handling system was critical to mend a failing heart. Studying cardiomyocyte maturation would be a necessary effort to develop such a therapeutic strategy.

Although the establishment of robust SERCA2 function and calcium transient is commonly treated as a hallmark of cardiomyocyte maturity, whether SERCA2 regulates cardiomyocyte maturation remain a major knowledge gap. Germline *Atp2a2* knockout mice were embryonic lethal, suggesting a critical role of SERCA2 in early heart development (Periasamy et al., 1999). By contrast, tamoxifen-inducible cardiomyocyte-specific depletion of SERCA2 in adult mice triggered only moderate heart dysfunction (Andersson et al., 2009), implying that the function of SERCA2 is heavily dependent on the stage of life. Therefore, it is necessary to directly define the role of SERCA2 in cardiomyocyte maturation, which is yet to be done in the field.

A Cas9/AAV9-mediated somatic mutagenesis system (CASAAV) was recently established for cardiomyocyte maturation studies (Guo et al., 2017; Vandusen et al., 2017). This technique harnessed adeno-associated virus seral type 9 (AAV9) to deliver the CRISPR/Cas9 system to efficiently create mutations specifically in neonatal cardiomyocytes in mice. In addition to its advantage in specifically targeting the maturation time window *in vivo*, CASAAV provided an easy means to create genetic mosaics in the heart (Guo and Pu, 2018), thereby studying the cell-autonomous function of the gene of interest without being complicated by animal death or the secondary effects of heart dysfunction (Guo et al., 2018; Guo et al., 2021).

In this study, we first built and validated the CASAAV system and next mutagenized *Atp2a2* specifically in neonatal cardiomyocyte in mice. In contrast to the moderate phenotypes in adult *Atp2a2* knockout mice*,* CASAAV-based *Atp2a2* knockout in neonates resulted in acute dilated cardiomyopathy and death within 3 weeks after AAV administration. In addition, the genetic mosaics analysis uncovered a global impact of SERCA2 on the ultrastructural and gene expression changes in cardiomyocyte maturation.

## 2 Materials and Methods

### 2.1 Animals

All animals and experiment protocols were approved by the Animal Ethical Committee of Peking University Health Science Center under the approval number LA2021034. Mice were housed at Peking University Health Science Center Department of Laboratory Animal Science. The homozygous Rosa^CAG−LSL-Cas9−2A-tdTomato^ mouse line was purchased from GemPharmatech (Strain No. T002249) (Chen et al., 2021).

In AAV injection, heart function analysis and tissue collection procedures, the mice were anesthetized with 2% isoflurane by inhalation, maintained asleep at 1.5% isoflurane, and euthanized through cervical dislocation if needed.

### 2.2 Plasmids and AAV Production

AAV-cTNT-Cre, AAV-U6sgRNA-U6sgRNA-cTNT-Cre, and AAV plasmids expressing sgRNAs targeting *Jph2*, *Tead1* and *Ryr2* were previously described (Guo et al., 2017). CRISPick (https://portals.broadinstitute.org/gppx/crispick/public) was used to design new sgRNAs targeting murine *Atp2a2*. The sequences of these sgRNAs were 5′-GCA​GAA​GCT​AGA​CGA​GTT​TG-3′ and 5′-CAA​GAT​CCG​GGA​TGA​AAT​GG-3’. To clone the AAV plasmid for *Atp2a2* knockout, the two sgRNA sequences were synthesized in primers and amplified together with the scaffold and U6 promoter sequences by PCR using 2xEasyPfu PCR SuperMix (-dye) (AS211-02, TransGen, Beijing, China). The PCR product was next incorporated into the AAV-U6sgRNA-U6sgRNA-cTNT-Cre plasmid between AarI and SapI sites using the seamless cloning master mix (B632219-0040, BBI, China).

AAV9 was prepared as previously described with modifications (Guo et al., 2017; Guo et al., 2018). In brief, 70 μg AAV plasmids, 70 μg AAV9-Rep/Cap and 160 μg pHelper (pAd-deltaF6, Penn Vector Core) plasmids were produced by the EZgeneTM Plasmid ezFilter Megaprep 3 Kit (PD1611-01, Biomiga) and transfected into five 15-cm plates of HEK293T cells using the PEI transfection reagent (40816ES03, Yeasen, China). At 60 h after transfection, cells were scraped off from the plates, resuspended in lysis buffer (20 mM Tris pH 8, 150 mM NaCl, 1mM MgCl_2_, 50 μg/ml benzonase (20156ES60, Yeasen, China)) and lysed by three freeze-thaw cycles. Simultaneously, AAV9 in the cell culture medium was precipitated by 40% PEG8000 (P2139, Sigma) in 2.5M NaCl solution, resuspended in lysis buffer and pooled with cell lysates. AAV was purified in an Optiprep (D1556-250ML, Sigma) density gradient by ultracentrifugation (XPN-100, Beckman) with a type 70Ti rotor. Next AAV was cleaned and concentrated in PBS with 0.001% pluronic F68 (PFL01-100ML, Caisson, China) using a 100 kDa centrifugal filter tube (UFC910096, Millipore). AAV titer was absolutely quantified by real-time PCR using primers amplifying a fragment of the cTNT promoter.

### 2.3 Echocardiogram and Electrocardiogram

Echocardiography was performed with a VINNO6n machine (VINNO Corporation, Suzhou, China). The mice were shaved on the chest and the videos were acquired with a 23 MHz transducer along the parasternal long axis. The Lead II electrocardiogram was measured with a Softron ECG processor and analyzed by SP2006 software (Softron Biotechnology, Beijing, China). The animal treatment was blinded to the scientist who performed the measurement. Five consecutive heart beats were averaged to calculate the results for each animal.

### 2.4 Reverse-transcription PCR (RT-PCR) and real-time quantitative PCR (RTqPCR)

Total RNA was purified from the heart apex using the TransZol Up Plus RNA Kit (ER501-01, TransGen, China). Genomic DNA removal and reverse transcription were performed using TransScript II One-Step gDNA Removal and cDNA Synthesis SuperMix (AH311-03, TransGen, China).

RT-PCR was performed using 2XTaq PCR Master Mix (KT211, TIANGEN). RT-PCR primers were designed as follow: 5′-TGC​CTT​GTT​GGG​GTG​ACT​G-3′ and 5′-CAC​CGT​TCC​ATT​GCT​GTG​C-3′ for *Jph2*; 5′-GCC​GTG​GTT​TGT​TTT​CTT​G-3′ and 5′-GCT​TGT​TGT​GGA​TGG​CAG​TAG-3′ for *Tead1*; 5′-TGA​GAA​CTG​ATG​ATG​AGG​TGG-3′ and 5′-GCT​TAG​AGG​CAG​GAT​GTA​TGG-3′ for *Ryr2*.

RT-qPCR was performed using Perfect Start Green qPCR Super Mix (+DveII) (AQ602-24, TransGen, China) and the AriaMx Real-Time PCR System (Agilent Technologies). RT-qPCR primers were: 5′-CAA​CTC​CCT​CAA​GAT​TGT​CAG​CAA-3′ and 5′-GGC​ATG​GAC​TGT​GGT​CAT​GA-3′ for *Gapdh*; 5′- GGT​AGC​CAA​TGC​AAT​CGT​GG-3′ and 5′-CGT​TGC​ACA​CTC​TTT​CTG​TCC-3′ for *Atp2a2*.

### 2.5 Western Blot

The fresh heart apex was washed by ice-cold PBS prior to homogenization with an electric homogenizer (DREMEL F6/10) in RIPA Buffer (25 mM Tris PH7.0∼8.0, 150 mM NaCl, 0.1% SDS, 0.5% sodium deoxycholate, 1% Triton X-100, protease inhibitors (Solarbio)). After 10-min lysis on ice, tissue lysates were centrifuged for 15 min at 12,000 rpm at 4°C. Then the supernatants were collected and the protein concentration was quantified using Easy II Protein Quantitative Kit (BCA) (DQ111-01, TransGen, Beijing, China). The protein concentration of the lysates was adjusted to the same level with RIPA Buffer and next diluted in 4x SDS sample buffer (P1016, Solarbio, China). After being boiled for 10 min, 20 μg proteins from each sample was separated on a 4–15% gradient gel (DG101-01, TransGen, China), transferred to a PVDF membrane and blocked for 1 h in 5% Non-Fat Milk/TBST at room temperature.

Primary antibodies (Ms-Anti-GAPDH, 1:5000, TransGen, HC301; Rb-Anti-SERCA2, 1:5000, Abcam, ab137020) were incubated with the membranes overnight at 4°C. After three washes with TBST, 5 min each, the membranes were incubated with HRP-conjugated secondary antibodies (HRP-Gt-Anti-Rb, 1:5000, D110058-0100, BBI; HRP-Gt-Anti-Ms, 1:5000, D110087-0100, BBI) for 1 h at room temperature. After three washes of TBST, 5 min each, the ECL Western blotting substrate (PE0010, Solarbio, China) was added to the membranes for chemiluminescence signal development. Images were acquired using an iBright CL1500 Imaging System (Thermo Fisher Scientific).

### 2.6 Cardiomyocyte Isolation and Confocal Imaging of Cardiomyocytes

Cardiomyocytes were isolated by retrograde Langendorff perfusion according to an established protocol (Guo et al., 2017; Guo et al., 2018). In brief, the mice were injected with heparin and anesthetized with isoflurane. Then the hearts were removed and cannulated through the aorta onto the perfusion apparatus. Perfusion buffer (120.4 mM NaCl, 14.7 mM KCl, 0.6 mM KH_2_PO_4_, 0.6 mM Na_2_HPO_4_, 1.2 mM MgSO_4_, 10 mM HEPES, 4.6 mM NaHCO_3_, 30 mM Taurine, 10 mM BDM, 5.5 mM Glucose) was applied to flushing blood and equilibrating the heart. After 4-min perfusion, the fluid was switched to myocyte digestion buffer containing 2 mg/ml collagenase II (Worthington, LS004177) for a 10-min digestion at 37°C.

After digestion, the apical half of heart was next cut off, manually dissociated into single cardiomyocytes in myocyte stopping buffer (10% FBS/perfusion buffer) and filtered through a 100 μm cell strainer. The cell suspension was concentrated by 20 g centrifugation for 5 min and re-suspended in short-term culture medium containing DMEM (Gibco), 10% FBS (SE100-B, VISTECH), Penicillin/Streptomycin (Gibco) and 10 μM Blebbistatin. Cardiomyocytes were cultured on laminin-coated coverslips for less than 1 h at 37°C with 5% CO2 before fixation.

For CellROX staining, we incubated the isolated cardiomyocytes with 5 μM CellROX Green Reagent (Life technologies, United States) for 10min before immediately imaging the cells using a confocal microscope. For *in situ* T-tubule imaging, hearts were perfused with perfusion buffer added with 1 μg/ml FM 4–64 (21487, AAT Bioquest) at room temperature for 10 min. After labeling, the heart was removed from the perfusion system, positioned on a glass-bottom dish, and immediately imaged using an inverted confocal microscope (Leica SP8).

### 2.7 Immunofluorescence

For immunofluorescence on isolated cardiomyocytes, the cells were fixed on coverslips by 4% paraformaldehyde/PBS for 10 min, permeabilized and blocked by 0.1% Triton-100 and 4% BSA/PBS at room temperature for 1 h. Primary antibodies (Ms-Anti-α-actinin, 1:500, abcam, ab9465) were incubated with the cells for 1 h at temperature. After 3-washes with PBS, 5 min each, fluorescent secondary antibodies (Alexa Fluor^®^ 488, 1:500, Abcam) and DAPI were incubated with the cells for 1 h at room temperature. After 3-washes with PBS, 5 min each, the cells were mounted with anti-fluorescence quenching mounting fluid (P0126-25ml, Beyotime) before confocal imaging.

For fluorescence detection on tissue sections, heart tissues were first immersed in 4% paraformaldehyde (PFA) at 4°C overnight and then dehydrated in sucrose solutions (15% followed by 30%) overnight. Then the tissue samples were embedded in OCT (Sakura) and frozen in -80 °C. Heart sections (7 μm) were cut on a cryostat microtome (CM 1950, Leica, Germany). For antibody staining, the tissue sections were incubated with primary antibodies (Rb-anti-Ki67, Cell Signaling Tech., 9129T) and then stained with the secondary antibodies. TUNEL staining was performed with the one-step fluorescence TUNEL kit (Beyotime, C1086). Or the sections were treated with wheat germ agglutinin (Alexa Fluor^®^ 488-conjugated, 1:500 dilution, AAT Bioquest) and DAPI for 10 min at room temperature. The slides were mounted with anti-fluorescence quenching mounting fluid (P0126-25ml, Beyotime) before confocal imaging.

The images were captured by a Leica SP8 confocal laser scanning microscope equipped with an APO 63X/1.4 oil objective. ImageJ was used for quantitative measurement. Cell size, cell shape and Z-line distance were manually measured.

### 2.8 Cell Sorting and RNA-Seq

Isolated cardiomyocytes were purified by a FACS Aria SORP (BD Biosciences) machine equipped with a 100 μm nozzle at Peking University Medical and Health Analysis Center. RNA was extracted from sorted cardiomyocytes with the RNAprep pure Micro Kit (Tiangen Biotech.). SMART-Seq was next performed at the Beijing Geekgene Company. First, the RNA was mixed with 0.1 μL rnase inhibitor (Clontech), 1.9 μL Triton X-100 solution (1%), 1 μL dNTP mix (10 mM), and 1 μL oligo-dT primer (5 μM). Reverse transcription was performed by further adding 0.5 μL SuperScript II reverse transcriptase (200 U/μL, Invitrogen), 0.25 μL rnase inhibitor (40 U/μL, Clontech), 2 μL Superscript II First-Strand Buffer (5×, Invitrogen), 0.5 μL DTT (0.1M, Invitrogen), 2 μL Betain (5 M, Sigma), 0.06 μL MgCl2 (1M, Sigma), and 0.1 μL TSO (100 μM). Reverse transcription was carried out at 25°C for 5 min, 42°C for 60 min, followed by 50°C for 30 min and 72°C for 10 min.

PCR preamplification was performed using KAPA HiFi HotStart Ready MIX (KAPA Biosystems) with 22 cycles of PCR and the IS PCR primer reduced to 50 nM (4 cycles at 98°C for 20 s, 65°C for 30 s, and 72°C for 5 min, followed by 18 cycles at 98°C for 20 s, 67°C for 15 s, and 72°C for 5 min, with a final cycle at 72°C for 5 min). Subsequently, Amplified samples were purified twice with 0.8X Ampure XP beads (Beckman, A63882).

Next, the enriched cDNA fragments were used to construct libraries using KAPA Hyper Prep Kits (KK8505). NEB U-shape adaptors were used for ligation. Libraries were sequenced to generate 150-bp paired-end reads on an Illumina X-Ten platform.

### 2.9 Statistical Analysis

The statistical analysis was performed using GraphPad software. Median, 25 and 75% quartiles (horizontal lines in the box), individual data points and maximal/minimal values (whiskers) were shown in the Box-and-Whisker plots. Unless otherwise stated, data were analyzed using unpaired two-tailed student’s t-test. *p* < 0.05 was considered statistically significant.

### 2.10 Data Availability

The sequencing data for this study has been deposited at the BIG submission portal, Genome Sequence Archive (CRA005866), and are accessible at https://ngdc.cncb.ac.cn/gsa/s/81v8l014. Other materials and data sets are available upon reasonable requests.

## 3 Results

### 3.1 Establishment and Validation of the CASAAV System

The conventional CASAAV system uses the Rosa^CAG−LSL-Cas9−2A−GFP^ mouse in which Cre recombinase can recombine the LoxP-STOP-LoxP (LSL) site and activate CAG promoter-driven Cas9-2A-GFP expression (Guo et al., 2017; Vandusen et al., 2017). Although this mouse was proved efficient in Cas9-based mutagenesis, the GFP marker was not only relatively dim but also precluded downstream assays using green fluorescence (Guo et al., 2018). To solve these problems, we adopted a newly generated Rosa^CAG−LSL-Cas9−2A-tdTomato^ mouse line (Rosa^Cas−Tom^) (Chen et al., 2021) in the CASAAV system, in which tdTomato is a red fluorescent protein that is much brighter than GFP (Shaner et al., 2004).

**FIGURE 1:**
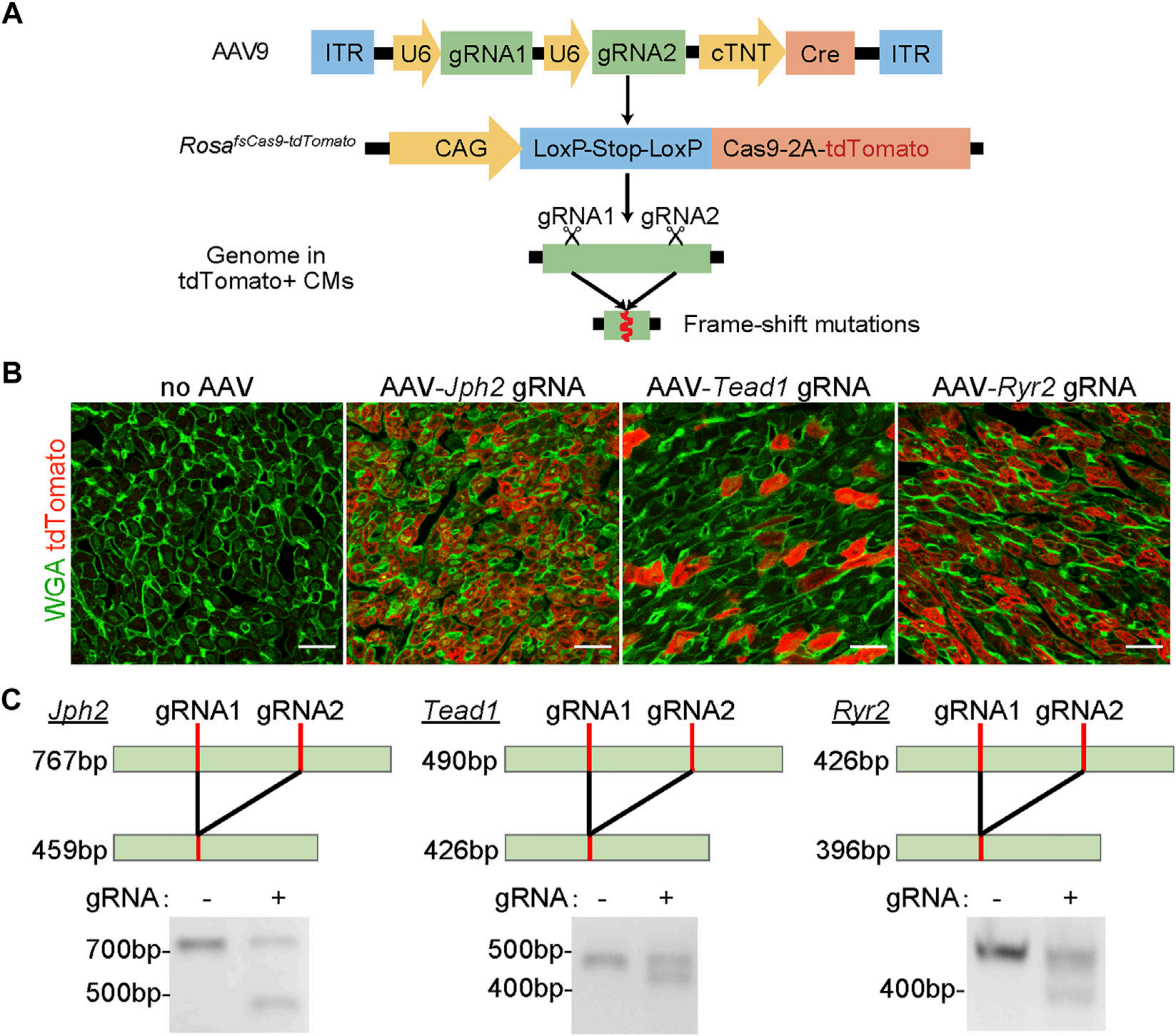
The validation of the CASAAV system. **(A)**, Diagram of the CASAAV working mechanism. In AAV9, U6 promoters drive the expression of sgRNAs. The cardiomyocyte-specific cTNT promoter drives Cre expression. Cre activates the expression of Cas9-2A-tdTomato in Rosa^Cas−Tom^ mice. Cas9-sgRNA complexes target the gene of interest by inducing frame-shift mutations. (**B)**, Confocal images of cardiac ventricular cryo-sections. The hearts were treated with AAVs previously validated in CASAAV experiments. WGA, wheat germ agglutinin. Scale bar, 20 μm. **(C)**, RT-PCR analysis of CASAAV targeted regions.

In the CASAAV system (Figure 1A), AAV vectors deliver Cre recombinase driven by a cardiomyocyte-specific cTNT promoter, thus Cas9-based mutagenesis is restricted to cardiomyocytes. The same AAV vectors also deliver two single-guide RNAs (sgRNAs) expressed by U6 promoters. The Cas9/sgRNA complexes target the coding sequences of the gene of interest, deposit small insertions and deletions to the genome, and eventually generate frame-shift mutations that silence the gene.

We first tested if the Rosa^Cas−Tom^ mice were suitable for CASAAV. We generated serotype-9 AAVs (AAV9) that were reported to efficiently knockout Jph2, Tead1 and Ryr2 in murine cardiomyocytes by CASAAV (Guo et al., 2017). We injected each AAV subcutaneously into homozygous postnatal day 1 (P1) Rosa^Cas−Tom^ pups at the dose of 5 × 10^10^vg/g (viral genome per Gram body weight). The heart ventricles were harvested at P7 for histological and molecular analysis. As compared to the control samples receiving no AAVs, all AAV-treated hearts exhibited bright red fluorescence (Figure 1B), indicating successful activation of the LSL and tdTomato components by the AAVs.

The sgRNAs in the above AAVs were purposely designed to target genomic regions proximal to each other (usually less than 300bp apart) so the DNA fragments between the two targeted loci would be deleted and readily detected by the size change of PCR products. We performed reverse-transcription PCR (RT-PCR) to amplify genomic regions including the sgRNAs targets. In control groups, single PCR products of the expected sizes were observed. However, in the AAV-treated groups, smaller PCR products corresponding to CASAAV-mediated DNA deletion were found (Figure 1C). These data indicate that CASAAV works in the Rosa^Cas−Tom^ mice.

### 3.2 Cardiomyocyte-specific Whole-Heart Analysis of *Atp2a2* Knockout Mice

**FIGURE 2:**
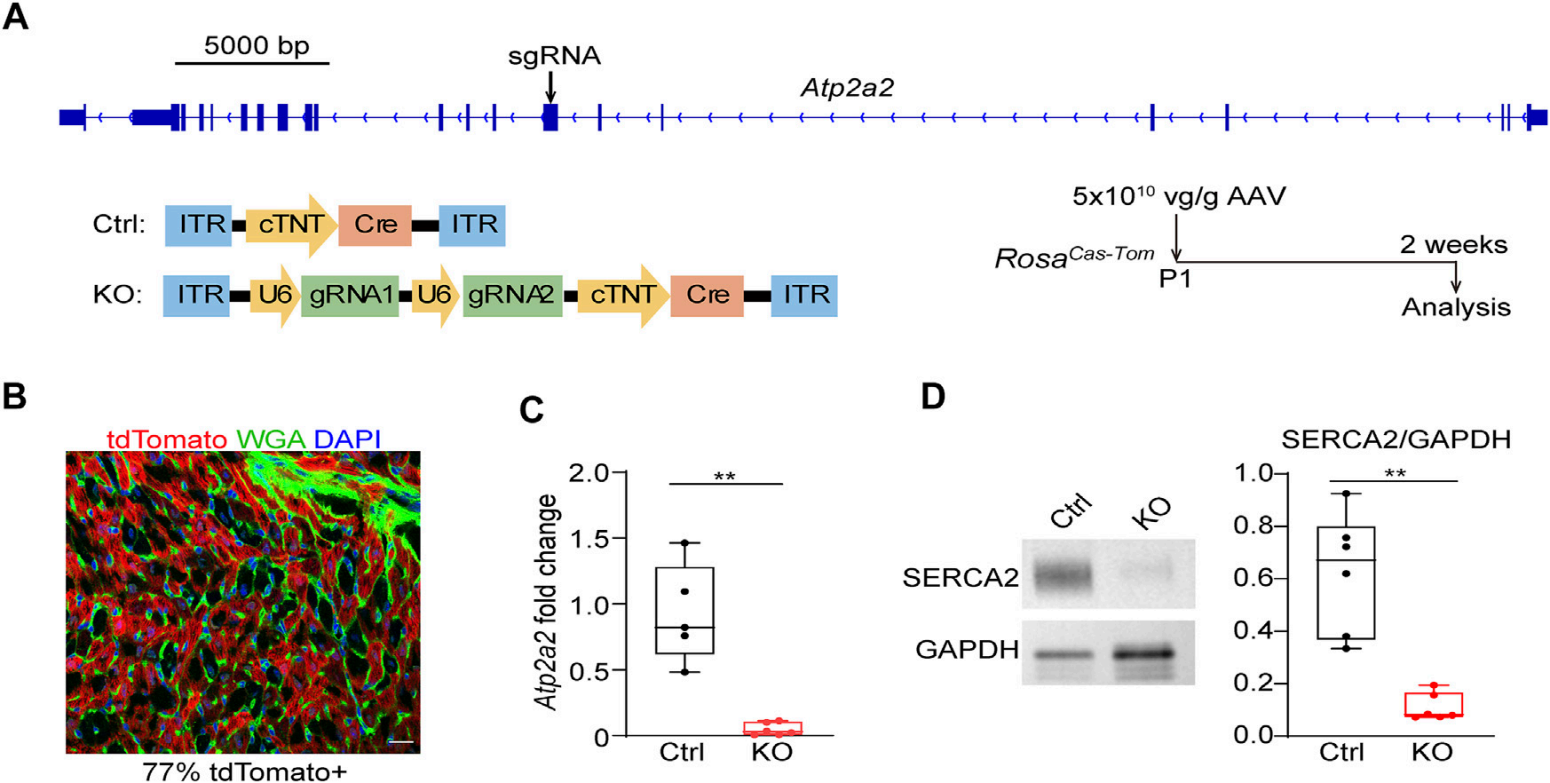
The establishment of CASAAV-based *Atp2a2* mutagenesis. **(A)**, Design of *Atp2a2-targeted* sgRNAs and CASAAV-based knockout experiments. **(B)**, A confocal image of the cardiac section treated with high-dose AAVs mediating *Atp2a2* knockout. Scale bar, 20 μm. **(C)**, Quantitative real-time PCR analysis of heart tissues treated with high-dose control and *Atp2a2* knockout AAVs. **(D)**, Western blot analysis and quantification of heart tissues treated with high-dose control and *Atp2a2* knockout AAVs. In **C** and **D**, Box and Whisker plots were presented to show individual data points (dots), 25%-50%–75% percentiles (horizontal lines in the boxes) and maximal/minimal values (whiskers). Unpaired two-tailed student’s t-test: **p < 0.01, ***p < 0.001.

We next designed sgRNAs targeting *Atp2a2* and prepared AAV9 vectors carrying these sgRNAs for CASAAV-based knockout (Figure 2A). AAV vectors that do not express sgRNAs were used as the control. We injected 5 × 10^10^ vg/g control and knockout vectors into P1 pups and collected heart tissues at P14 to determine *Atp2a2* knockout efficiency. Fluorescence imaging analysis of heart sections stained with wheat germ agglutinin (WGA), a marker of cell boundary, revealed 77% AAV transduction rate among cardiomyocytes (Figure 2B), thus we considered this “high-dose” AAV treatment a whole-heart experiment suitable for tissue level analysis. Real-time quantitative PCR (RT-qPCR) analysis showed profound reduction of *Atp2a2* mRNA in the CASAAV-treated heart tissues (Figure 2C), which led to the reduction of SERCA2 protein levels as shown by western blot analysis (Figure 2D). To directly validate the Cas9-based genome mutagenesis, we performed RNA-Seq analysis on the AAV-transduced cardiomyocytes (see Section 3.4 for more details). We found the number of sequencing reads aligned to *Atp2a2* exons was greatly reduced, presumably due to non-sense mediated mRNA decay (Supplementary Figure S1A). Among reads aligned to the sgRNA-targeted exon, we observed insertion and deletion mutations adjacent to the sgRNA targets, directly supporting the action of Cas9-based mutagenesis (Supplementary Figure S1B). Together, these data indicate the successful establishment of cardiomyocyte *Atp2a2* knockout in mice.

**FIGURE 3:**
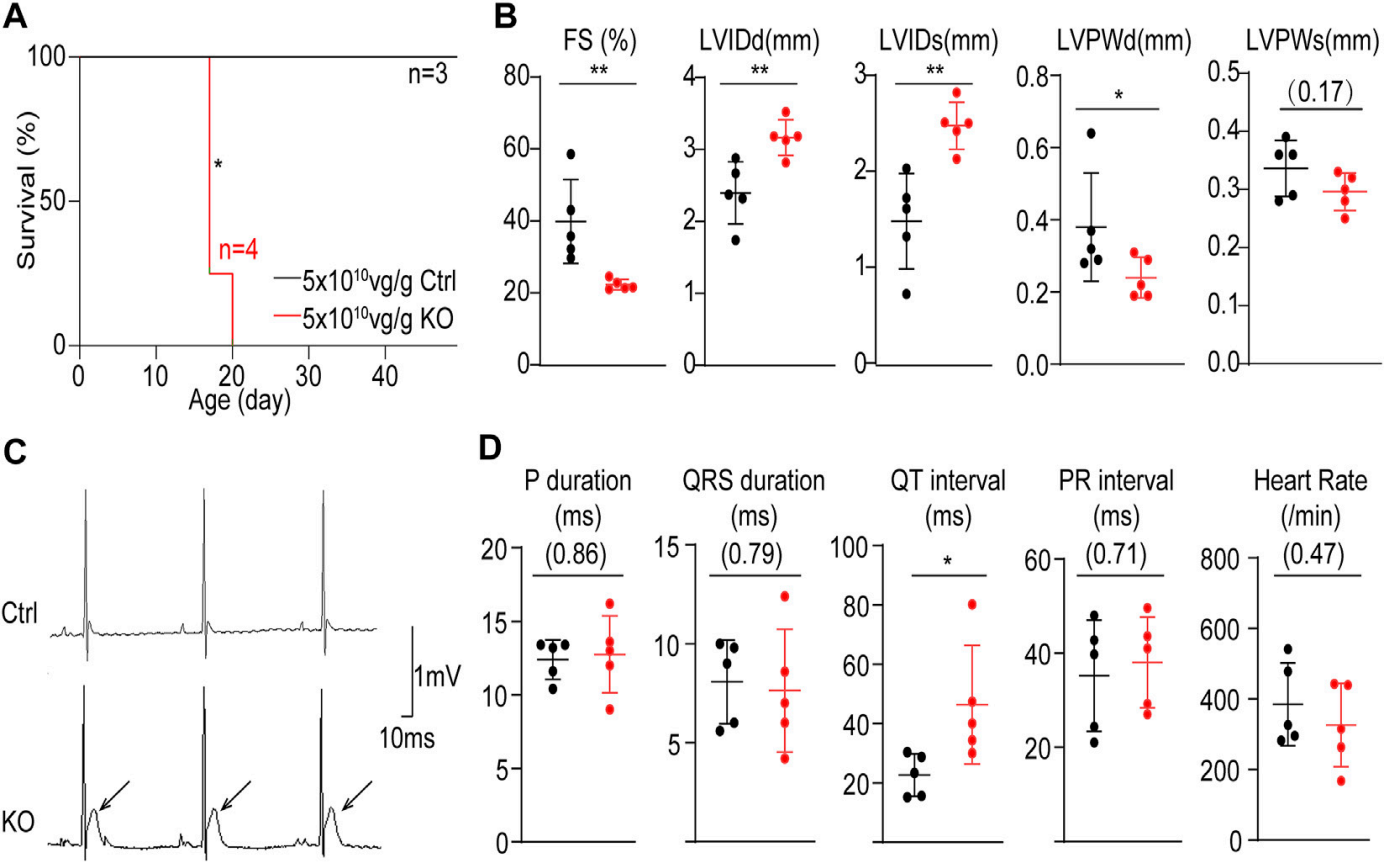
The impact of whole-heart cardiomyocyte-specific *Atp2a2* knockout on cardiac functions. **(A)**, Survival analysis of animals treated with high-dose control (Ctrl) and *Atp2a2* knockout (KO) AAVs. Log-rank test, *p < 0.05. **(B)**, Quantification in echocardiogram analysis. FS, fractional shortening; LVIDd, left ventricular internal diameter end diastole; LVIDs, left ventricular internal diameter end systole; LVPWd, left ventricular posterior wall end diastole; LVPWs, left ventricular systolic posterior wall end systole. **(C)**, Representative electrocardiogram traces. Arrows point to elevated ST segments. **(D)**, Quantification in electrocardiogram analysis. In **C** and **D**, mean ± SD (horizontal lines) and individual data points (dots) were presented. Unpaired two-tailed student’s t-test: *p < 0.05, **p < 0.01, non-significant *p* values in parenthesis.

We next assessed the impact of whole-heart *Atp2a2* knockout on heart functions. The high dose-treated P1-injected mice undergoing *Atp2a2* CASAAV knockout died in the third week after birth (Figure 3A). Echocardiogram analysis demonstrated reduced fraction shortening (FS), increased left ventricular internal diameters at the end of diastole (LVIDd) and systole (LVIDs), as well as a decrease of left ventricular posterior wall thickness at the end of diastole (LVPWd) (Figure 3B; Supplementary Figure S2) in the P14 mutant hearts. Thus, whole-heart *Atp2a2* knockout led to reduced cardiac contractile functions and ventricular dilatation.

We also performed electrocardiogram analysis and observed prominent ST segment elevation in P14 mutant hearts (Figure 3C). This phenotype was specifically associated with increased QT intervals while no other measured electrocardiac parameters were changed (Figure 3D). Therefore, *Atp2a2* knockout perturbed cardiac electrophysiology particular in the ventricular repolarization phase.

### 3.3 Genetic Mosaic Analysis of *Atp2a2* Knockout Cardiomyocytes

The prominent phenotypes observed in the P14 mutant hearts strongly implied the presence of cardiomyocyte maturation defects. However, the whole-heart cardiac dysfunction could deposit secondary effects that confound further cell maturity analysis (Guo et al., 2017; Guo and Pu, 2018). To solve this problem, we next titrated down AAV doses and performed a genetic mosaic analysis.

**FIGURE 4:**
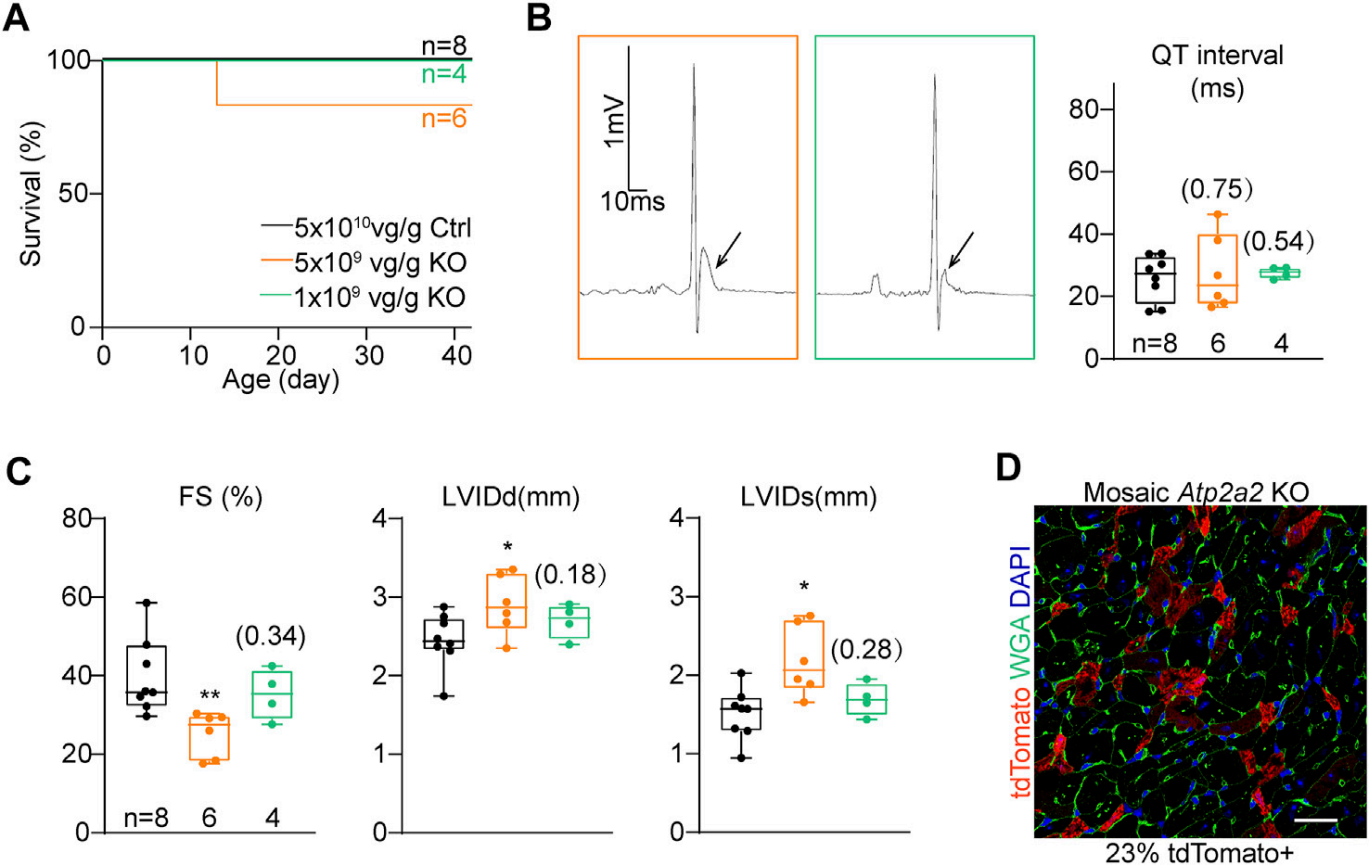
The titration of AAV dosages for genetic mosaic analysis. **(A)**, Survival analysis of animals treated with high-dose control (Ctrl) and titrated *Atp2a2* knockout (KO) AAVs. **B**, Representative electrocardiogram traces of animals with titrated AAV doses and the relevant QT interval quantification. Arrows point to ST segments. **(C)**, Echocardiogram analysis of animals with titrated AAV doses. In **(B)** and **C**, box and whisker plots were presented to show individual data points (dots), 25%-50%–75% percentiles (horizontal lines in the boxes) and maximal/minimal values (whiskers). Unpaired two-tailed student’s t-test: *p < 0.05, **p < 0.01, non-significant *p* values in parenthesis. (**D)**, A representative image of ventricle sections from hearts treated with the low-dose AAV. Scale bar, 20 μm.

We serially diluted the knockout AAV and injected 5 × 10^9^ vg/g and 1 × 10^9^ vg/g to P1 Rosa^Cas−Tom^ pups. These two treatments were 1:10 and 1:50 diluted as compared to the high-dose treatment, and thus referred as “mid-dose” and “low-dose” treatments in the following experiments. In both treatments, animal deaths were circumvented within the cardiac maturation time window (Figure 4A). The ST segment elevation phenotypes were attenuated in the mid-dose treatment and barely detectable in the low-dose group (Figure 4B). Low-dose AAV treated *Atp2a2* knockout hearts displayed nearly normal contractile functions and ventricle sizes (Figure 4C; Supplementary Figure S2). In this experiment, fluorescence imaging revealed about 23% tdTomato positive cardiomyocytes in the heart sections (Figure 4D). Thus, we successfully generated a genetic mosaic model to study *Atp2a2* knockout cardiomyocytes *in vivo* without the concern that the phenotypes were secondary to overall cardiac dysfunction. This model was next used in all following assays.

**FIGURE 5:**
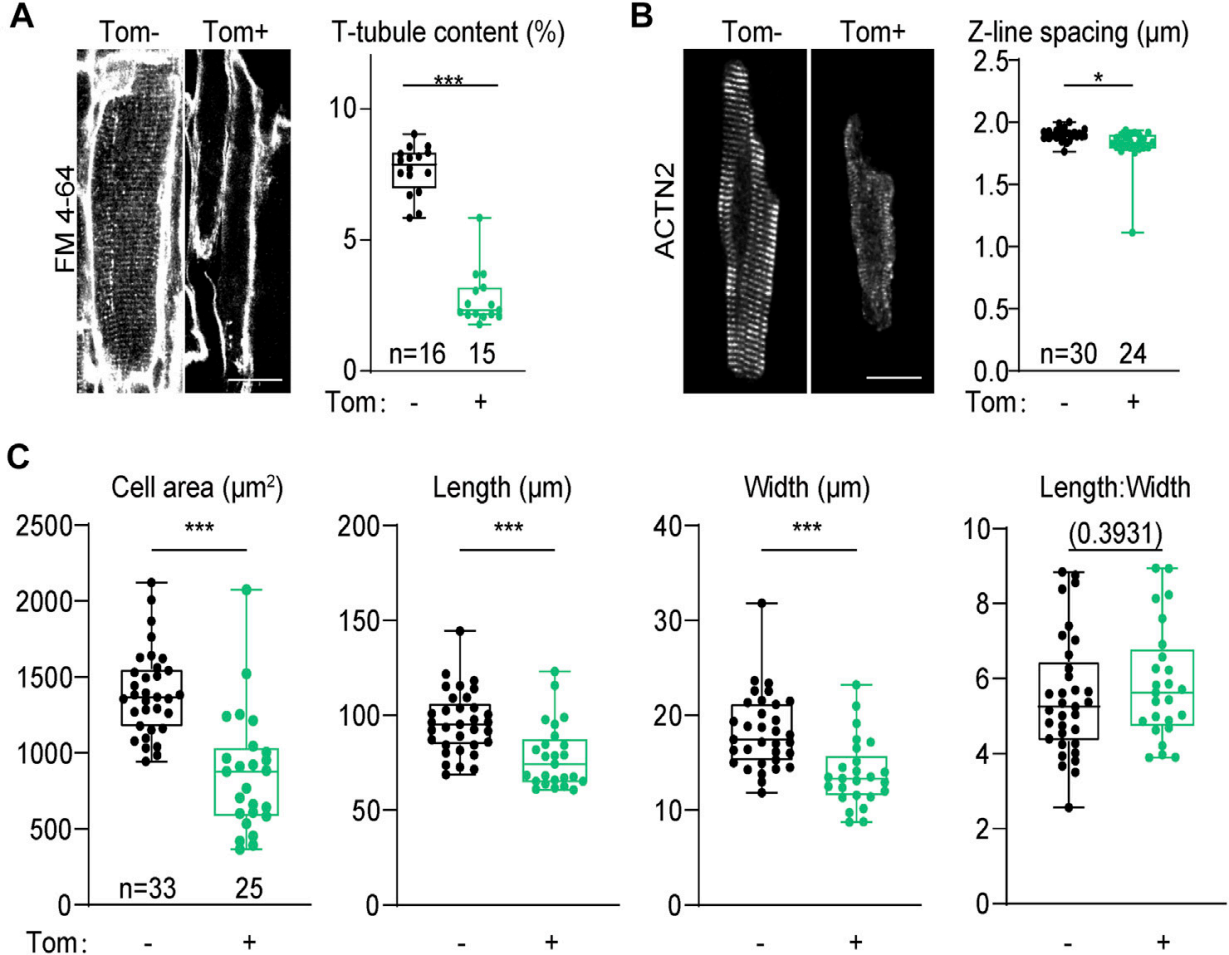
Morphological analysis of cardiomyocytes from the genetic mosaic hearts**. (A)**, *In situ* confocal images of cardiomyocytes stained with FM 4–64 and AutoTT quantification of T-tubule contents. **(B)**, Confocal images of isolated cardiomyocytes immunolabeled with an ACTN2 antibody and quantification of Z-line distance. In **A** and **B**, scale bar, 20 μm. **(C)**, Geometric measurement of isolated cardiomyocytes. Tom, tdTomato. Unpaired two-tailed student’s t-test: *p < 0.05, ***p < 0.001, non-significant *p* values in parenthesis.

At the cellular level, we first wondered if SERCA2 regulated T-tubule, the key ultrastructural hallmark of calcium handling maturation. We harnessed Langendorff retrograde perfusion to load the whole heart with a cell membrane dye FM 4–64 and performed confocal optical section to directly observe T-tubule *in situ* (Chen et al., 2015). This experiment was performed on 4-week old hearts when T-tubule maturation just finished (Chen et al., 2013). In tdTomato-negative cardiomyocytes that were not transduced by AAV, transversely-aligned T-tubules could be observed throughout the cytoplasm. However, in tdTomato-positive *Atp2a2* knockout cardiomyocytes, no sign of T-tubule formation was detected (Figure 5A). AutoTT software (Guo and Song, 2014) was used to quantify T-tubule contents and corroborated the observation (Figure 5A).

Sarcomere expansion is another hallmark of cardiomyocyte maturation. To determine the impact of *Atp2a2* knockout on sarcomere expansion, we isolated cardiomyocytes from 4-week-old mosaic hearts and immunolabeled sarcomeres with the Z-line marker ACTN2 (α-actinin-2). This work demonstrated reduced ACTN2 signals in the mutant cardiomyocytes as well as the decreased spacing between adjacent Z-lines (Figure 5B). We also measured the projected cell area, cell length and cell width and we found these parameters were all reduced in *Atp2a2* knockout cardiomyocytes (Figure 5C). However, the length: width ratios of the mutant cardiomyocytes were maintained, thus SERCA2 promoted the hypertrophic growth of cardiomyocytes by proportionally increasing cell length and width.

### 3.4 Transcriptomic Assessment of the Cell-Autonomous *Atp2a2* Functions

**FIGURE 6:**
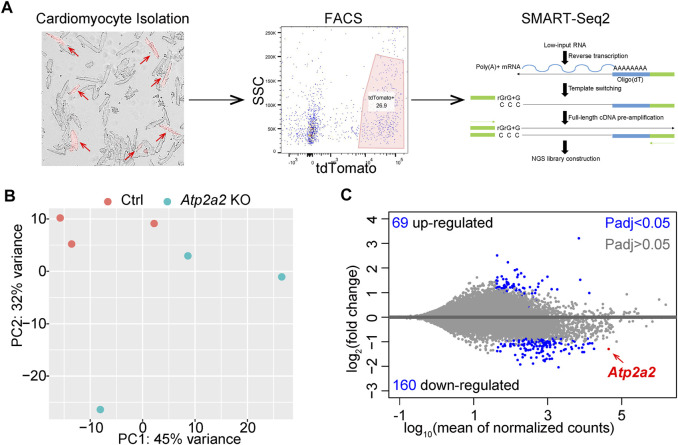
RNA-Seq analysis of FACS-enriched cardiomyocytes. **(A)**, A diagram of FACS-RNA-Seq workflow. Representative images of isolated cardiomyocytes, flow cytometry gating, and the principle of SMART-Seq2 are shown. Red arrows point to tdTomato + cardiomyocytes. **(B)**, PCA plot of individual RNA-seq data. **(C)**, MA plot of differential expression analysis. **B** and **C** were generated with the DESeq2 software.

In addition to the above structural parameters, cardiomyocyte maturation was also characterized by profound gene expression changes (Guo and Pu, 2020). To investigate the impact of *Atp2a2* knockout on cardiomyocyte maturation, we next performed RNA sequencing analysis. The genetic mosaic approach adds technical difficulties in enriching AAV-treated cardiomyocytes for bulk analysis. To solve this problem, we developed a protocol to perform fluorescence-activated cell sorting (FACS) of isolated cardiomyocytes from mice and extracted the small amount of mRNA from the sorted cells (Guo et al., 2018). We also adopted the SMART-Seq (Switching Mechanism At the end of the 5′-end of the RNA Transcript) technique (Picelli et al., 2013) to perform transcriptomic analysis from the low input of mRNA (Figure 6A).

We analyzed three biological replicates of control and *Atp2a2* knockout cardiomyocytes that were collected at P14 and observed clear separation between the two groups of data in a principal component analysis (PCA) (Figure 6B). Using adjusted *p* value (Padj) less than 0.05 as the cutoff, we identified 69 upregulated genes and 160 down-regulated genes, among which *Atp2a2* counts were one of the most significantly reduced (Figure 6C). Thus, this FACS and SMART-Seq platform provided a reliable method to uncover major transcriptional changes in cardiomyocytes and to understand the cell-autonomous functions of SERCA2 in cardiomyocyte maturation.

**FIGURE 7:**
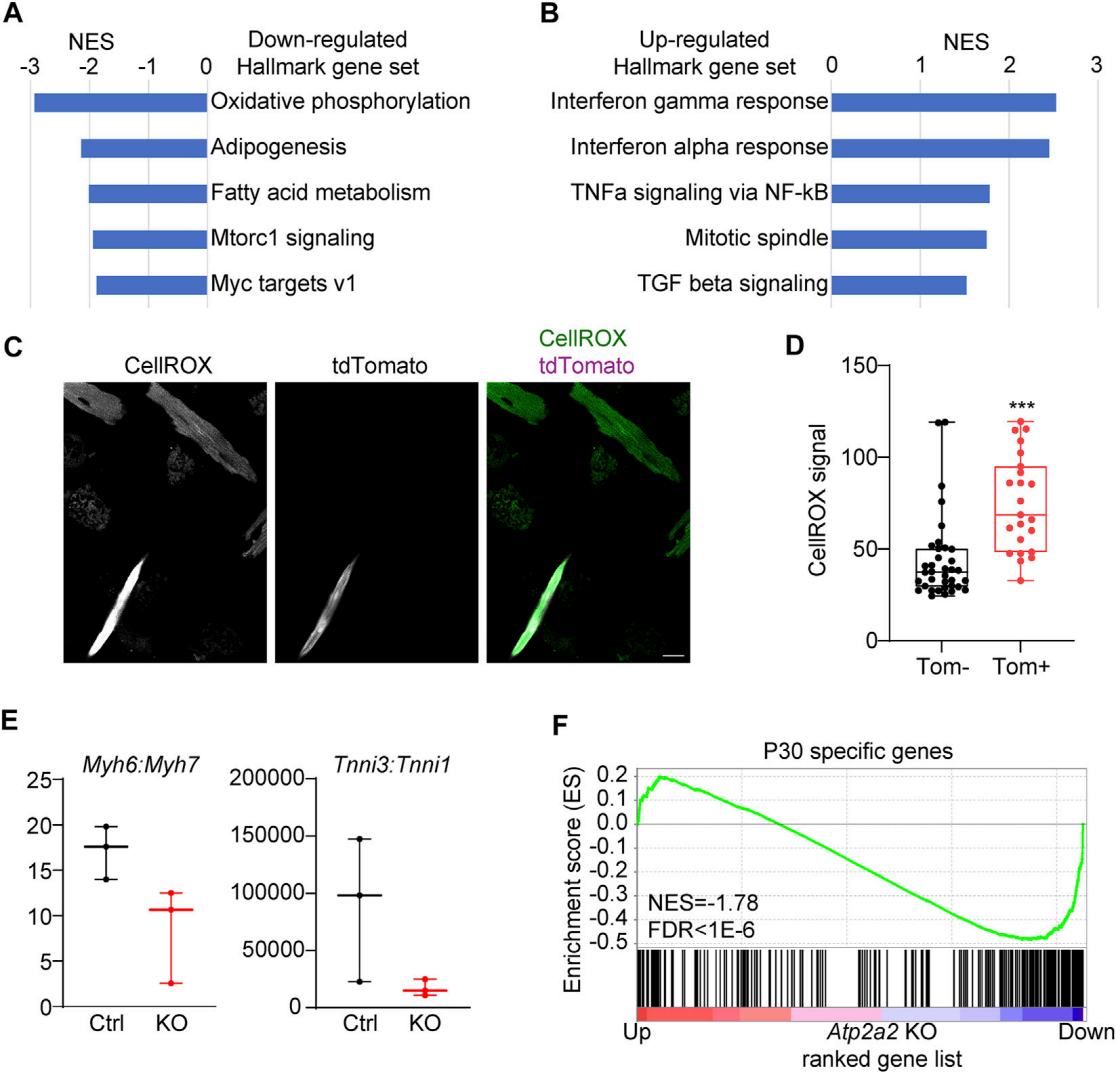
Transcriptomic maturation defects in Atp2a2-deleted cardiomyocytes. **(A-B)**, GSEA of the top dysregulated Hallmark gene sets in *Atp2a2*-deleted cardiomyocytes. **(C-D),** CellRox staining and quantification of ROS in isolated cardiomyocytes. Scale bar, 20 μm. Unpaired two-tailed student’s t-test: ***p < 0.001. **(E)**, RPKM ratio analysis of *Myh6-7* and *Tnni3-1* isoform switching. **(F)**, GSEA of P30-specific genes in *Atp2a2*-deleted cardiomyocytes.

We performed gene set enrichment analysis (GSEA) to uncover the major biological functions associated with the differentially regulated genes in the *Atp2a2* knockout cardiomyocytes. Among the Hallmark gene sets, oxidative phosphorylation and fatty acid metabolism genes appeared most enriched in the down-regulated genes (Figure 7A). This data indicated cardiomyocyte maturation defects since oxidative respiration using fatty acids as substrates was a major metabolic feature for mature cardiomyocytes (Guo and Pu, 2020). Inflammation-related gene sets such as Interferon and TNFa responses were up-regulated (Figure 7B). Both oxidative respiratory and inflammatory changes were known to associated with reactive oxygen species (ROS) elevation. Agreeing to this notion, we stained isolated cardiomyocytes with a ROS dye CellROX and observed a significant increase of the ROS signal in the *Atp2a2* mutant cells (Figure 7C,D).

A hallmark of murine cardiomyocyte maturation is the isoform switching from the fetal expression of *Myh7* and *Tnni1* to the adult expression of *Myh6* and *Tnni3* (Hunkeler et al., 1991; Ng et al., 1991). In the SERCA2-depleted cardiomyocytes, we observed decreased Myh6:Myh7 and Tnni3:Tnni1 ratios (Figure 7E), further supporting the presence of cardiomyocyte maturation defects. Another important hallmark of cardiomyocyte maturation is the withdrawal from cell cycle. Thus, we performed GSEA of the cell proliferation gene set and observed weak but significant enrichment in the upregulated genes in the SERCA2-depleted cardiomyocytes (Supplementary Figure S3A). However, immunostaining of Ki67 in the *Atp2a2* mutant cardiac sections did not show increase of proliferation in the AAV-infected tdTomato + cardiomyocytes (Supplementary Figure S3B, arrow heads). To assess gene expression changes at the transcriptome level, we harnessed a published RNA-Seq data comparing P6 vs P30 cardiomyocytes using the FACS-SMART-Seq experimental setup identical to this study (Guo et al., 2021). GSEA revealed P30-specific genes were significantly enriched among the down-regulated genes in the *Atp2a2* knockout cardiomyocytes (Figure 7F). Thus, SERCA2 globally regulated the transcriptome changes during cardiomyocyte maturation.

Because calcium is well-known to regulate cardiomyocyte survival versus death, thus we also examined the impact of SERCA2 depletion on cell death. We performed GSEA and observed significant upregulation of a cell death gene set in the *Atp2a2* knockout cardiomyocytes (Supplementary Figure S3C). The terminal deoxynucleotidyl transferase dUTP nick-end labeling (TUNEL) technique further validated the presence of dying cardiomyocytes in the high-dose AAV-treated *Atp2a2* mutant hearts (Supplementary Figure S3D), while no such signal was detected in the control heart sections. This result suggested that the cardiomyocyte maturation defects of the *Atp2a2* knockout cardiomyocytes were partly associated with cell death.

## 4 Discussion

SERCA2 is a central regulator of calcium handling in cardiomyocytes. The reduction of SERCA2 is responsible for the progression of heart failure after a variety of cardiac injuries. Elevating SERCA2 function has been the first and major effort in the gene therapy clinical trials for heart failure (Jessup et al., 2011; Zsebo et al., 2014; Greenberg et al., 2016). Despite the pivotal role of SERCA2 as a therapeutic target, little is known about its role in cardiomyocyte maturation, largely due to the lack of an appropriate technical platform for this purpose.

In this study, we achieved Cas9/AAV9-based *Atp2a2* knockout specifically in neonatal maturing cardiomyocytes in mice. We found whole-heart *Atp2a2* knockout through high-dose AAV administration triggered cardiac dysfunction including the reduced contractility, enlarged left ventricle, ST segment elevation and animal death. These phenotypes are more severe than those observed in adult-specific *Atp2a2* knockout animals (Andersson et al., 2009), implying a new maturation-specific role of this gene that could not be simply explained by calcium homeostasis functions.

Through careful titration of AAV dosage, we created a genetic mosaic model to circumvent animal death and cardiac dysfunction that were associated conventional whole-heart SERCA2 depletion (Periasamy et al., 1999; Andersson et al., 2009). We uncovered a series of novel, profound, and cell-autonomous functions of SERCA2 in cardiomyocyte maturation, including regulating T-tubule, sarcomeres, cellular hypertrophy, oxidative respiration and gene expression. This discovery highlighted the power of genetic mosaic analysis in the effort to precisely dissect gene functions that would otherwise be confounded by secondary effects of cardiac dysfunction.

A major unanswered question in this study pertains to the mechanisms by which *Atp2a2* knockout led to the observed phenotypes. Elevated cytoplasm calcium is known to activate calcineurin-NFAT and calmodulin-dependent protein kinase II (CaMKII) pathways in cardiomyocytes. However, calcineurin activation is known to promote cardiomyocyte hypertrophy (Molkentin et al., 1998), which is contradictory to the reduced size of *Atp2a2* knockout cardiomyocytes observed in this study. We analyzed CaMKII phosphorylation in the *Atp2a2* knockout cardiomyocytes but was not able to detect obvious activation of this kinase (data not shown). Together, the classic calcium signaling pathways are unlikely the main cause of phenotypes in the *Atp2a2* knockout phenotypes.

We observed dysregulated genes regarding oxidative respiration and TNFa responses by RNA-Seq. These data suggested metabolic or inflammatory changes might underlie the maturation defects of *Atp2a2* knockout cardiomyocytes. However, we currently are not able to narrow down the differentially regulated genes to examine the hypothesis in a candidate-based study. Instead, we plan to establish a recently published CASAAV-based forward genetic screen (Vandusen et al., 2021) to systemically study the link between SERCA2 and cardiomyocyte maturation in the following studies.

Heart failure is in many ways characterized by decreased cardiomyocyte maturity, a phenomenon commonly called dedifferentiation (Szibor et al., 2014). Ultrastructurally, heart failure hallmarks include less aligned sarcomeres, regressed T-tubules and fragmented mitochondria. Transcriptionally, heart failure-related cardiomyocyte dedifferentiation is characterized by the reactivation of fetal gene expression programs (Dirkx et al., 2013). SERCA2-based cardiac therapy is conventionally built on the theory that SERCA2 activation could enhance calcium handling performance. Based on this study, we propose that SERCA2 exerts additional therapeutic impact on the failing heart by promoting or maintaining cardiomyocyte maturity. This new insight should prompt the field to put more effort on cardiomyocyte maturation research as a mean to uncover new therapeutic targets for heart failure.

## Data Availability

The datasets presented in this study can be found in online repositories. The names of the repository/repositories and accession number(s) can be found in the article/Supplementary Material.
