# Supplementary material for: Cas9/AAV9-Mediated Somatic Mutagenesis Uncovered the Cell-Autonomous Role of Sarcoplasmic/Endoplasmic Reticulum Calcium ATPase 2 in Murine Cardiomyocyte Maturation
Source: Front Cell Dev Biol. 2022 Apr 1;10:864516. doi: 10.3389/fcell.2022.864516 (PMC9012521; doi:10.3389/fcell.2022.864516)
Supplement: Supplementary file 1 [file DataSheet2.PDF]

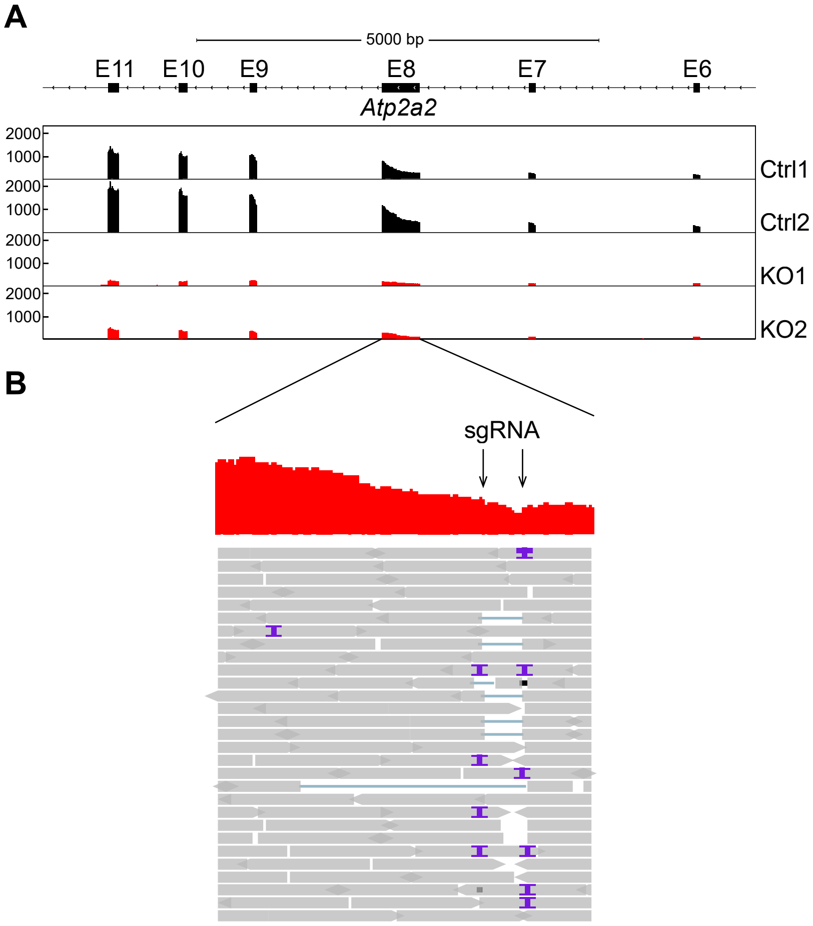

Supplementary Figure 1. Sequencing analysis of CASAAV-based *Atp2a2* mutagenesis. A, RNA-Seq reads distribution on *Atp2a2* exon 6-11. E, exon. **B,** an enlarged view of the RNA reads aligned to the a mutated exon by CASAAV. Representative reads aligned to this exon were shown below. Gaps in the reads showed small deletion mutations. Purple lines marked small insertion mutations. This plot was generated by the integrated genome viewer (IGV).
