# Supplementary material for: Cas9/AAV9-Mediated Somatic Mutagenesis Uncovered the Cell-Autonomous Role of Sarcoplasmic/Endoplasmic Reticulum Calcium ATPase 2 in Murine Cardiomyocyte Maturation
Source: Front Cell Dev Biol. 2022 Apr 1;10:864516. doi: 10.3389/fcell.2022.864516 (PMC9012521; doi:10.3389/fcell.2022.864516)
Supplement: Supplementary file 2 [file DataSheet4.PDF]

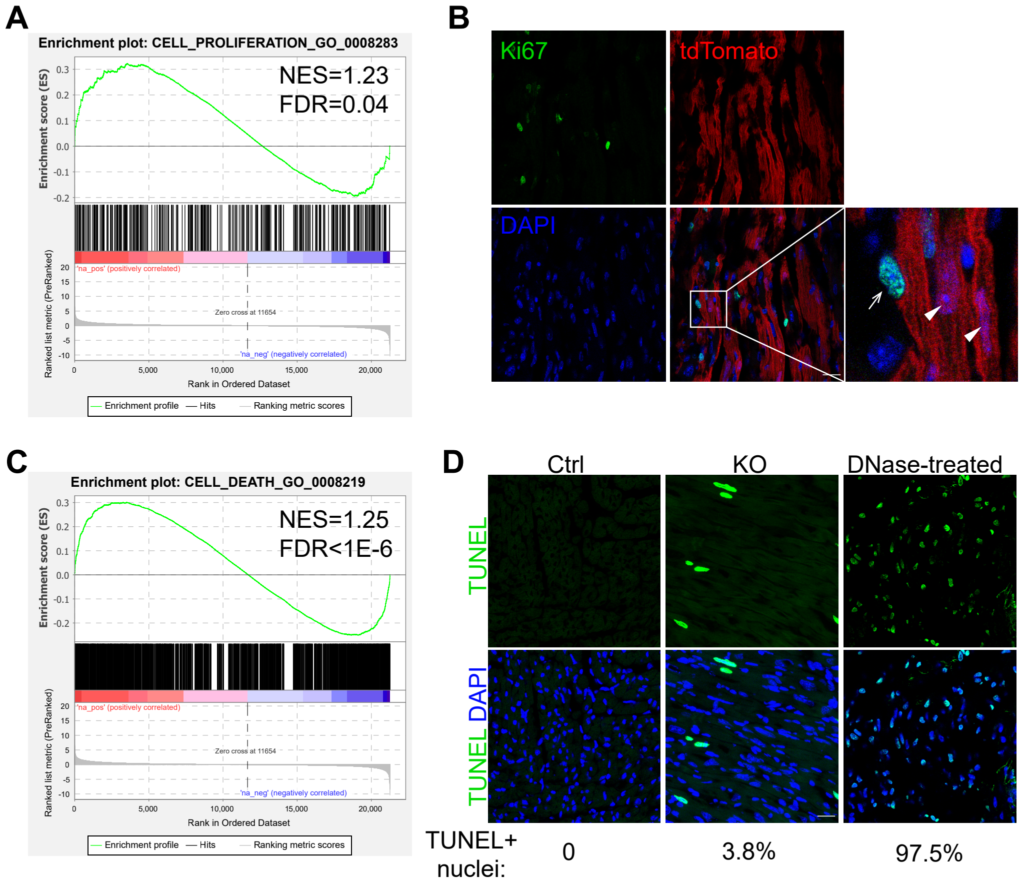

Supplementary Figure 3. The impact of CASAAV-based *Atp2a2* mutagenesis on cell proliferation and cell death. A, GSEA of *Atp2a2* knockout RNA-Seq data on cell proliferation. **B,** immunostaining of Ki67 on a high-dose AAV-treated *Atp2a2* knockout heart section. Scale bar, 20μm. The arrow points to a tdTomato-;Ki67+ nucleus; the arrowheads point to tdTomato+;Ki67- nuclei. **C,** GSEA of *Atp2a2* knockout RNA-Seq data on cell death. **D,** TUNEL staining on control and high-dose AAV-treated *Atp2a2* knockout heart sections. Scale bar, 20μm. The DNase-treated section as a positive control.
