# Supplementary material for: Cas9/AAV9-Mediated Somatic Mutagenesis Uncovered the Cell-Autonomous Role of Sarcoplasmic/Endoplasmic Reticulum Calcium ATPase 2 in Murine Cardiomyocyte Maturation
Source: Front Cell Dev Biol. 2022 Apr 1;10:864516. doi: 10.3389/fcell.2022.864516 (PMC9012521; doi:10.3389/fcell.2022.864516)
Supplement: Supplementary file 3 [file DataSheet3.PDF]

	Control	High -dose KO	Mid -dose KO	Low -dose KO
EF/%	71.94 ±2.88	51.88 ±1.78***	56.07 ±11.20**	71.18 ±8.64
FS/%	39.22 ±6.91	22.28 ±1.46**	25.18 ±5.73**	36.24 ±6.39
LVIDd/mm	2.42 ±0.21	3.17 ±0.25**	2.90 ±0.38*	2.72 ±0.19
LVIDs/mm	1.59 ±0.27	2.48 ±0.25**	2.19 ±0.45*	1.79 ±0.26
LVPWd/mm	0.30 ±0.15	0.24 ±0.06*	0.26 ±0.05	0.28 ±0.01
LVPWs/mm	0.34 ±0.08	0.30 ±0.03	0.30 ±0.02	0.32 ±0.04
LVAWd/mm	0.29 ±0.09	0.25 ±0.04	0.24 ±0.02	0.25 ±0.03

Supplementary Figure 2. Additional panels of echocardiogram analysis upon CASAAV-based *Atp2a2* mutagenesis. EF, ejection fraction; FS, fractional shortening; LVIDd, left ventricle internal diameter end diastole; LVIDs, left ventricle internal diameter end systole; LVPWd, left ventricle posterior wall thickness end diastole; LVPWs, left ventricle posterior wall thickness end systole; LVAWd, left ventricle anterior wall thickness end diastole. Unpaired two-tailed student's t-test: *P<0.05, **P<0.01, ***P<0.001.
